# Inhibitory effect of the reversal agents V-104, GF120918 and Pluronic L61 on MDR1 Pgp-, MRP1- and MRP2-mediated transport

**DOI:** 10.1054/bjoc.2000.1260

**Published:** 2000-07-03

**Authors:** R Evers, M Kool, A J Smith, L van Deemter, M de Haas, P Borst

**Affiliations:** Division of Molecular Biology and Center for Biomedical Genetics, The Netherlands Cancer Institute, Plesmanlaan 121, Amsterdam, CX, 1066, The Netherlands; Georg-Speyer-Haus, Institute for Biomedical Research, Paul Ehrlich Straße 42–44, Frankfurt a.M., 60596, Germany; Department of Neurology, Academic Medical Center, Amsterdam, AZ, 1105, The Netherlands; Department of Gastroenterology and Liver Diseases, Academic Medical Center, Amsterdam, AZ, 1105, The Netherlands

**Keywords:** multidrug resistance, reversal agent, MRP1, multispecific organic anion transporter, MDR1 Pgp, polarized cell

## Abstract

The human multidrug transporter MDR1 P-glycoprotein and the multidrug resistance proteins MRP1 and MRP2 transport a range of cytotoxic drugs, resulting in multidrug resistance in tumour cells. To overcome this form of drug resistance in patients, several inhibitors (reversal agents) of these transporters have been isolated. Using polarized cell lines stably expressing human *MDR1*, *MRP1* or *MRP2* cDNA, and 2008 ovarian carcinoma cells stably expressing *MRP1* cDNA, we have investigated in this study the specificity of the reversal agents V-104 (a pipecolinate derivative), GF120918 (an acridone carboxamide derivative also known as GG918), and Pluronic L61 (a (poly)oxypropethylene and (poly)oxypropylene block copolymer). Transport experiments with cytotoxic drugs with polarized cell lines indicate that all three compounds efficiently inhibit MDR1 Pgp. Furthermore, V-104 partially inhibits daunorubicin transport by MRP1 but not vinblastine transport by MRP2. V-104 reverses etoposide resistance of 2008/MRP1 cells, whereas GF120918 does not reverse resistance due to MRP1. V-104 partially inhibits the export of the organic anion dinitrophenyl *S* -glutathione by MDCKII-MRP1 but not by MDCKII-MRP2 cells. Unexpectedly, export of the organic anion calcein by MDCKII-MRP1 and MDCKII-MRP2 cells is stimulated by Pluronic L61, probably because it relieves the block on entry of calcein AM into the cell by endogenous MDR1 Pgp. © 2000 Cancer Research Campaign


*ttttt*(2000) **83**(3), 366–374
© 2000 Cancer Research Campaign
doi: 10.1054/ bjoc.2000.1260, available online at http://www.idealibrary.com on IDEAL®

### Inhibitory effect of the reversal agents V-104, GF120918 and Pluronic L61 on MDR1 Pgp-, MRP1- and MRP2-mediated transport

**R Evers[1,2], M Kool[1,3], AJ Smith[1,4], L van Deemter[1], M de Haas[1] and P Borst[1]**

[1]The Netherlands Cancer Institute, Division of Molecular Biology and Center for Biomedical Genetics, Plesmanlaan 121, 1066 CX Amsterdam, The Netherlands; [2]Georg-Speyer-Haus, Institute for Biomedical Research, Paul Ehrlich Straße 42–44, 60596 Frankfurt a.M., Germany; [3]Current address Academic Medical Center, Department of Neurology, 1105 AZ Amsterdam, The Netherlands; [4]Current address Academic Medical Center, Department of Gastroenterology and Liver Diseases, 1105 AZ Amsterdam, The Netherlands

**Summary** The human multidrug transporter MDR1 P-glycoprotein and the multidrug resistance proteins MRP1 and MRP2 transport a range of cytotoxic drugs, resulting in multidrug resistance in tumour cells. To overcome this form of drug resistance in patients, several inhibitors (reversal agents) of these transporters have been isolated. Using polarized cell lines stably expressing human *MDR1*, *MRP1* or *MRP2* cDNA, and 2008 ovarian carcinoma cells stably expressing *MRP1* cDNA, we have investigated in this study the specificity of the reversal agents V-104 (a pipecolinate derivative), GF120918 (an acridone carboxamide derivative also known as GG918), and Pluronic L61 (a (poly)oxypropethylene and (poly)oxypropylene block copolymer). Transport experiments with cytotoxic drugs with polarized cell lines indicate that all three compounds efficiently inhibit MDR1 Pgp. Furthermore, V-104 partially inhibits daunorubicin transport by MRP1 but not vinblastine transport by MRP2. V-104 reverses etoposide resistance of 2008/MRP1 cells, whereas GF120918 does not reverse resistance due to MRP1. V-104 partially inhibits the export of the organic anion dinitrophenyl *S*-glutathione by MDCKII-MRP1 but not by MDCKII-MRP2 cells. Unexpectedly, export of the organic anion calcein by MDCKII-MRP1 and MDCKII-MRP2 cells is stimulated by Pluronic L61, probably because it relieves the block on entry of calcein AM into the cell by endogenous MDR1 Pgp. © 2000 Cancer Research Campaign

**Keywords**: multidrug resistance; reversal agent; MRP1; multispecific organic anion transporter; MDR1 Pgp; polarized cell

Simultaneous resistance of tumour cells against a range of cytotoxic drugs is a serious problem in cancer chemotherapy. This phenomenon is called multidrug resistance (MDR). One form of MDR can be caused by members of the ATP-binding cassette (ABC) family of transport proteins (Gottesman et al, 1995; Cole and Deeley, 1998). These are large polytopic membrane proteins that actively transport drugs out of the cell, resulting in a decreased intracellular drug concentration. In humans, two ABC transporters have been identified that can cause resistance in tumour cells: MDR1 P-glycoprotein (Pgp) (Juliano and Ling, 1976), and the multidrug resistance protein (MRP1) (Cole et al, 1992). MDR1 Pgp transports drugs in an unmodified form, whereas MRP1 transports drugs either conjugated to the anionic ligands glutathione (GSH), glucuronide, or sulphate (Leier et al, 1994; Müller et al, 1994; Jedlitschky et al, 1997), or transports them in an unmodified form, probably together with GSH (Rappa et al, 1997; Loe et al, 1998).

Another human ABC transporter that might contribute to drug resistance is the liver canalicular multispecific organic anion transporter (cMOAT or MRP2). Studies with mutant rats (TR–/GY or EHBR) that lack the MRP2 protein in the canalicular membrane of the hepatocytes and with cell lines stably transfected with *MRP2* cDNA have demonstrated that the substrate specificity of this transporter is similar to that of MRP1, and suggested that MRP2 is able to transport several anti-cancer drugs (e.g. Evers et al, 1998; Masuda et al, 1998; Chen et al, 1999; Cui et al, 1999; Hooijberg et al, 1999). Besides MRP1 and MRP2, at least four other MRP homologs have been identified: MRP3, MRP4, MRP5 and MRP6 (Kool et al, 1997; 1999*a*). MRP3 has been shown to transport organic anions and confers low-level resistance against etoposide and tenoposide, and high-level resistance against methotrexate (Hirohashi et al, 1999; Kool et al, 1999*b*). Recently, a correlation was found between the overexpression of MRP4 and resistance against nucleoside-based antiviral drugs (Schuetz et al, 1999).

The potential involvement of the overproduction of drug pumps in clinical drug resistance in tumour cells has led to a search for compounds that can be used to inhibit these transporters in cancer patients. These so-called reversal agents or modulators should preferably be: i) Selective for one or more transporters and bind to these transporters with a high affinity, ii) non-toxic, and iii) stable in human plasma (Sarkadi and Müller, 1997). Several compounds have been described that effectively block MDRI Pgp-mediated drug resistance, some of which are currently being tested in the clinic (e.g. Fisher and Sikic, 1995; Rowinsky et al, 1998). Examples of such inhibitors are the non-immunosuppressive cyclosporin A analogue PSC833 (Twentyman and Bleehen, 1991), the pipecolinate derivative VX710 (Germann et al, 1997*a*; Yanigazawa et al, 1999), the acridone carboxamide derivative GF120918 (Hyafil et al, 1993), and the (poly)oxypropethylene and (poly)oxypropylene block copolymers Pluronic L61 and P85 (Venne et al, 1996; Miller et al, 1997; Batrakova et al, 1998).

*Received 6 January 2000*
*Revised 28 March 2000*
*Accepted 3 April 2000*

*Correspondence to:* R Evers (Georg-Speyer-Haus)

Recently, we established two model systems suitable for testing inhibitors of MDR1 Pgp, MRP1 and MRP2: i) A set of polarized cell lines stably expressing *MDR1*, *MRP1*, or *MRP2* cDNA (Schinkel et al, 1995; Evers et al, 1996; 1998). In these cells MDR1 Pgp and MRP2 are localized in the apical plasma membrane, whereas MRP1 is localized in the lateral plasma membrane. The ability of these cell lines to grow in a monolayer make them suitable for drug-transport studies and for determining the specificity and efficiency of inhibitors (Evers et al, 1996; 1998; Smith et al, 1998); ii) 2008 ovarian carcinoma cells stably expressing various *MRPs* (Hooijberg et al, 1999; Kool et al, 1999*b*).

To further explore the suitability of transfected (polarized) cell lines for the screening of reversal agents and to investigate the specificity of previously identified reversals, we describe in this report the effect of the inhibitors Pluronic L61, V-104 (an analogue of VX710), and GF120918 on MDR1 Pgp-, MRP1- and MRP2-mediated transport.

### MATERIALS AND METHODS

### Materials

[$^3$H]Vinblastine (15 Ci mmol$^{-1}$), [$^{14}$C]-1-chloro-2,4-dinitrobenzene ([$^{14}$C]CDNB; 10 Ci mol$^{-1}$), and inulin-[$^{14}$C] carboxylic acid (5.95 Ci mol$^{-1}$) were obtained from Amersham Pharmacia Biotech (Little Chalfont, UK). [$^3$H]Daunorubicin (4 Ci mmol$^{-1}$) was from NEN Life Science Products (Boston, MA, USA). Calcein acetoxymethyl (AM) ester was from Molecular Probes (Leiden, The Netherlands), Pluronic L61 from Supratek Pharma Inc. (Laval, Canada), V-104 ((S)-1-[2-Oxo-2-(3,4,5-trimethoxy-phenyl)acetyl]piperidine-2-carboxylic acid (R and S)-1-(3-phenyl-propyl)-4-pyridin-3-yl-butyl ester; also called VRT-010367) from Vertex Pharmaceuticals Inc. (Cambridge, MA, USA), and GF 120918 from GlaxoWellcome (Greenford, UK). Other chemicals and drugs were from Sigma Chemical Co. (St Louis, MO, USA).

### Cell lines

Polarized canine (MDCKII) or porcine (LLC-PK1) kidney-cell lines stably expressing *MDR1*, *MRP1*, or *MRP2* (*cMOAT*) have been described before (Schinkel et al, 1995; Evers et al, 1997; 1998; Bakos et al, 1998). MDCKII-derived cell lines were cultured in DMEM with 10% fetal calf serum, LLC-PK1-derived cell lines in Medium 199 with 10% fetal calf serum. 2008/MRP1 cells have been described before (Hooijberg et al, 1999), and were cultured in RPMI 1640 with 10% fetal calf serum.

### Transport assays

[$^3$H]vinblastine and [$^3$H]daunorubicin transport assays were carried out essentially as described (Evers et al, 1996). Cells were seeded on microporous polycarbonate filters (3 µm pore size, 24.5 mm diameter, Transwell™ 3414; Costar Corp., Cambridge, MA, USA) and cultured for 3 days with a daily medium replacement. The experiment was started ($t$ = 0) by replacing the medium at either the apical or the basal side of the cell layer with 2 ml of complete medium containing 2 µM of drug (at 0.25 µCi ml$^{-1}$), [$^{14}$C]-labelled inulin (0.025µ Ci ml$^{-1}$), and the amount of inhibitor indicated. The cells were incubated at 37°C, 5% $CO_2$. 50 µl aliquots were taken from each compartment at various time-points. Radioactivity was measured as the fraction of radioactivity added at the beginning of the experiment. The paracellular flux was monitored by the appearance of inulin [$^{14}$C]carboxylic acid in the opposite compartment and was typically below 1.5% per h. Inhibition of transport by inhibitors was calculated by dividing the net flux of drug in the control experiment (at $t$ = 4 h) by the net flux determined in the presence of inhibitor. Export of [$^{14}$C]DNP-GS was determined by incubating cells with 2 µM of [$^{14}$C]CDNB (15 nCi ml$^{-1}$) at room temperature as described previously (Evers et al, 1996; 1998), with the modification that the experiments were performed in Hanks Balanced Salt Solution (containing 1.3 mM $CaCl_2$). If inhibitors were used in the experiment, cells were first incubated with HBSS containing inhibitor for 10 min before adding the substrate. Export of calcein was determined by incubating monolayers in HBSS containing calcein AM (1 µM) at room temperature. Samples (200 µl) were taken at the time-points indicated. 800 µl HBSS was added and fluorescence was determined in a fluorimeter (Perkin-Elmer). The amount of calcein exported was calculated using a calibration curve made with free calcein.

All transport experiments were performed in duplicate and repeated at least twice.

### Cytotoxicity assays

The drug sensitivity of cells was determined as described (Kool et al, 1999*b*) in growth inhibition assays with continuous exposure to drug.

### RESULTS

### Inhibition of drug transport by Pluronic L61

To examine whether Pluronic L61 inhibits transport mediated by MDR1 Pgp, vectorial transport of vinblastine was measured in LLC-MDR1 cells in the presence of various Pluronic L61 concentrations. Figure 1 shows that transport was blocked by this compound; total inhibition of vinblastine transport was already obtained at a Pluronic L61 concentration of 0.04% (w/v). No effect on the paracellular [$^{14}$C]inulin flux was observed at concentrations up to 0.1% (w/v) Pluronic L61 (results not shown). To investigate whether Pluronic L61 could inhibit vinblastine transport by MRP2, we transfected MDCKII cells (Evers et al, 1998), as MRP2-mediated transport was very low in transfected LLC-PK1 cells (RE and PB, unpublished data). In these experiments a low concentration of the Pgp inhibitor PSC833 (0.1 µM) was present to inhibit the endogenous Pgp in these cells. This concentration of PSC833 only partially inhibits MDR1 Pgp in MDCKII-MDR1 cells and has no effect on vinblastine transport by MDCKII-MRP2 cells. At the highest concentration Pluronic L61 (0.1% (w/v)) tested, some inhibition (1.5-fold) of net vinblastine transport by MDCKII-MRP2 cells was observed, whereas transport mediated by MDR1 Pgp was completely blocked under these conditions (Figure 2). At lower concentration of Pluronic L61 (0.04% (w/v)), transport by MRP2 was not affected at all, whereas transport by MDR1 Pgp was totally blocked (data not shown).

The effect of Pluronic L61 on MRP1 is summarized in Table 1. In polarized cells MRP1 routes to the basolateral membrane (Evers et al, 1996) and not to the apical membrane, like Pgp and MRP2.

© 2000 Cancer Research Campaign

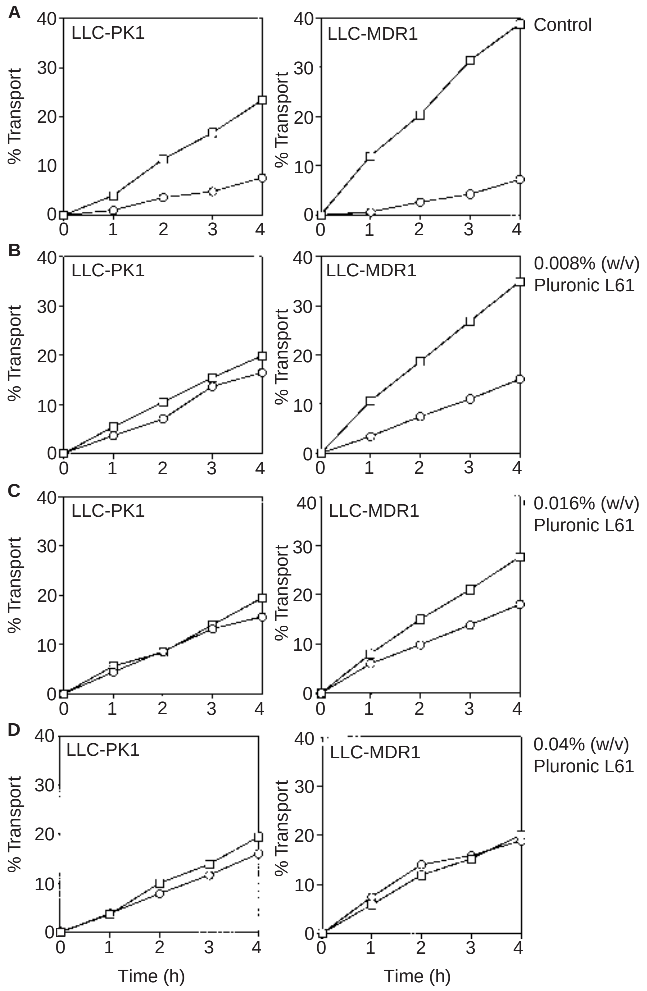

**Figure 1** Effect of Pluronic L61 on vinblastine transport by LLC-PK1 and LLC-MDR1 cells. (**Left graphs**) LLC-PK1 wild-type cells. At *t* = 0 [$^3$H]vinblastine (2 μM) was applied either to the apical or basolateral compartment, and the amount of radioactivity appearing in the opposite compartment was determined. Transport is presented as the fraction of total radioactivity added at the beginning of the experiment, appearing in the opposite compartment. (**Right graphs**) Same as left graphs but LLC-MDR1 cells were tested. Transport was tested in the absence of Pluronic L61 (**A**), or in the presence of 0.008% (w/v) (**B**), 0.016% (w/v) (**C**), or 0.04% (w/v) Pluronic L61 (**D**). Squares: translocation from basolateral to apical. Circles: translocation from apical to basolateral. Experiments were performed in duplicate. Variation between measurements was between the size of the symbols

Moreover, MRP1 does not increase transport of vinblastine through kidney-cell monolayers (Evers et al, 1996), and we therefore studied transport of dinitrophenol *S*-glutathione ([$^{14}$C]DNP-GS) instead (Oude Elferink et al, 1993; Evers et al, 1996; 1998). DNP-GS does not enter the cells, but cells do take up the precursor [$^{14}$C]CDNB, which is converted intracellularly into DNP-GS by glutathione *S*-transferases (GSTs). Table 1 shows that Pluronic L61 has a small inhibitory effect on the basolateral [$^{14}$C]DNP-GS export by MDCKII-MRP1 cells, but also on apical export by MDCKII-MRP2 cells. As the total amount of DNP-GS retrieved in medium and cells was somewhat lower in the presence of Pluronic L61 in all cell lines (Table 1), it is probable that Pluronic L-61 had some inhibitory effect on the formation of DNP-GS rather than directly on MRP.

Calcein acetoxy-methyl ester (calcein AM) is a lipophilic compound that enters the cell by passive diffusion over the plasma membrane. Cleavage of the ester bonds by intracellular esterases converts calcein AM into the highly fluorescent organic anion calcein. Calcein AM itself is efficiently transported by MDR1 Pgp (Homolya et al, 1996), and MRP1 transports both calcein AM and calcein (Feller et al, 1995; Holló et al, 1996). As transport of fluorescent substrates can be conveniently measured using a fluorimeter, we investigated whether calcein could be used as a substrate to study the function of MRP1 and MRP2 in MDCKII cells. Cells were incubated in the presence of calcein AM and the amount of calcein appearing in the medium was determined. Figure 3A shows that in MDCKII wild-type cells calcein export to the apical and basolateral side was similar. Hardly any calcein export was detected in MDCKII-MDR1 cells (Figure 3B), some increased (2-fold) basolateral export was observed in MDCKII-MRP1 cells (Figure 3C), and a 3-fold increased apical export was measured in MDCKII-MRP2 cells (Figure 3D). Unexpectedly, in the presence of Pluronic L-61 calcein export to the basolateral side by the MDCKII-MRP1 cells was increased 2-fold (*t* = 2 h), and export to the apical side by the MDCKII-MRP2 cells was increased 4-fold. Similar data were obtained in the presence of higher Pluronic L61 concentrations (data not shown). An obvious explanation for this paradoxical stimulation is that Pluronic L61 inhibits the endogenous MDR1 Pgp present in MDCKII cells and that this Pgp limits the entry of calcein AM in the absence of Pluronic L61. To test this hypothesis, calcein transport was measured in the presence of PSC833 (0.1 μM). This concentration of PSC833 had some stimulatory effect on export by MDCKII-MRP2 cells, but no effect on export by MDCKII-MRP1 cells (Figure 3C and 3D).

### Inhibition of drug transport by V-104

VX710 is a potent inhibitor of MDR1 Pgp in several drug-selected cell lines that overexpress *MDR1* (Germann et al, 1997*a*). In addition, VX710 partially reverses the MDR phenotype of *MRP1* overexpressing cells (Germann et al, 1997*b*; Yanagisawa et al, 1999). An analogue of VX710 has been developed called V-104. At 15 μM, V-104 effectively inhibited daunorubicin and vinblastine transport by MDCKII-MDR1 cells (Figures 4B and 5B). Hardly any inhibition of MDR1 was observed at lower concentrations of V-104 (5 μM) (data not shown). V-104 at 15 μM had a modest inhibitory effect on MRP1. The low daunorubicin transport by MDCKII-MRP1 shown in Figure 4C was somewhat decreased by the drug. DNP-GS export (Table 1) was decreased as well. Note that the experiments with MDCKII-MRP1 cells in Table 1 show that V-104 decreases basolateral DNP-GS transport by nearly 30%, whereas endogenous DNP-GS goes up 2-fold. As substrate (DNP-GS) production could be limiting to some extent in these cells, the 30% inhibition is a minimal value. In contrast, vinblastine transport by MDCKII-MRP2 cells was not affected by V-104 (Figure 5C). Table 1 shows that export of DNP-GS from MDCKII-MRP2 cells was not significantly affected by V-104 either.

### Inhibition of drug transport by GF120918

GF120918 is an efficient inhibitor of MDR1 Pgp (Hyafil et al, 1993; Wallstab et al, 1999). Figures 4B and 5B show that GF120918 completely inhibited daunorubicin and vinblastine

 © 2000 Cancer Research Campaign

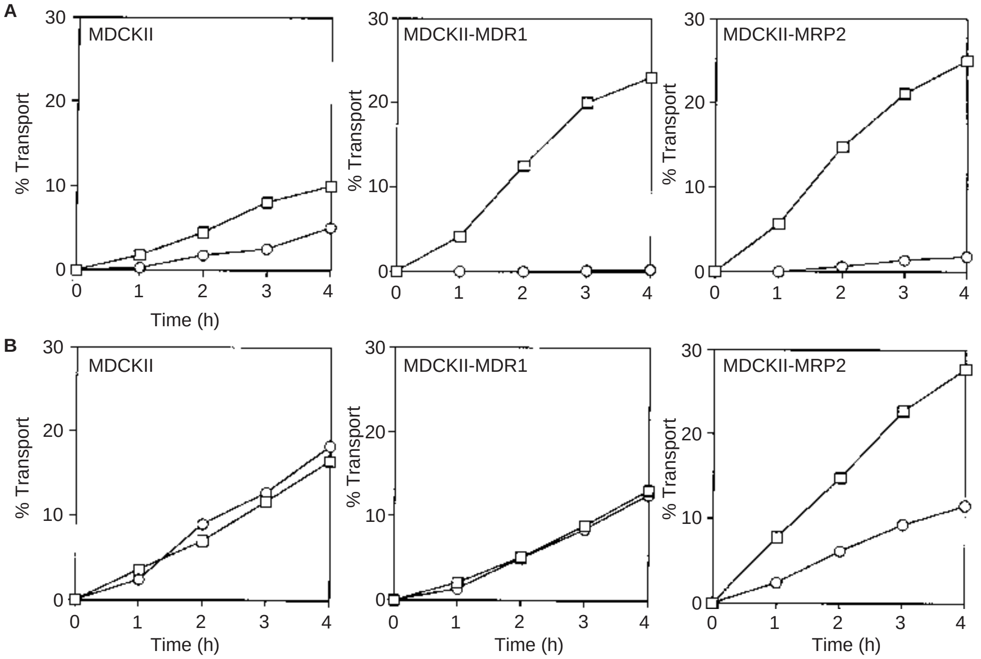

**Figure 2** Effect of Pluronic L61 on vinblastine transport through polarized MDCKII cell monolayers. Transport of [$^3$H]vinblastine was performed as in Figure 1, but with PSC833 (0.1 μM) present in the apical and basal compartment during all experiments. (**A**) Transport in MDCKII, MDCKII-MDR1 and MDCKII-MRP2 cells, respectively. (**B**) Same as **A**, but transport was determined in the presence of Pluronic L61 (0.1% (w/v)). Squares: translocation from basolateral to apical. Circles: translocation from apical to basolateral

**Table 1** Effect of various inhibitors on DNP-GS transport by MDCKII cell monolayers

| Cell line | MDCKII | | | | MDCKII-MRP1 | | | | MDCKII-MRP2 | | | |
|---|---|---|---|---|---|---|---|---|---|---|---|---|
| inhibitor | Apical | Basal | Intracellular | Total | Apical | Basal | Intracellular | Total | Apical | Basal | Intracellular | Total |
| Control | 490 ± 19 | 855 ± 6 | 1456 ± 10 | 2801 | 185 ± 5 | 2113 ± 61 | 136 ± 0 | 2434 | 1670 ± 43 | 506 ± 4 | 315 ± 8 | 2491 |
| Pluronic L61 (0.1%) | 393 ± 17 | 1167 ± 10 | 861 ± 0 | 2401 | 147 ± 6 | 1818 ± 12 | 131 ± 11 | 2096 | 1396 ± 13 | 450 ± 7 | 239 ± 23 | 2085 |
| V-104 (15 μM) | 737 ± 39 | 767 ± 12 | 806 ± 2 | 2301 | 196 ± 18 | 1497 ± 26 | 290 ± 6 | 1983 | 1765 ± 13 | 440 ± 3 | 186 ± 5 | 2391 |
| GF120918 (5 μM) | 616 ± 17 | 1200 ± 41 | 796 ± 27 | 2612 | 218 ± 0 | 2246 ± 29 | 166 ± 1 | 2630 | 1728 ± 27 | 570 ± 27 | 225 ± 17 | 2523 |

Cells growing in a monolayer were preincubated in the presence of the inhibitors indicated for 10 min. At $t = 0$ [$^{14}$C]CDNB (final concentration 2 μM; 15 nCi ml$^{-1}$) was added to both the apical and basal compartment. Samples were taken at $t = 1$, 3, 6, 12, and 20 min and extracted with ethyl acetate. The experiments were performed in duplicate and the variation between the measurements is indicated. Values presented represent the total amount of [$^{14}$C]DNP-GS (in pmoles) formed after 20 min.

transport by MDCKII-MDR1 cells, whereas transport of daunorubicin by MDCKII-MRP1 cells (Figure 4C) and vinblastine transport by MDCKII-MRP2 cells (Figure 5C) was not affected. Table 1 shows that export of DNP-GS by MDCKII-MRP1 and MDCKII-MRP2 cells was not inhibited by GF120918 either.

### Reversal of drug resistance by V-104 and GF120918

To test whether the reversal agents GF120918 and V-104 were able to reverse the drug resistance of 2008/MRP1 cells, we performed growth inhibition assays with continuous drug exposure with or without inhibitor. The maximum concentrations of the inhibitors that were not cytotoxic during a continuous exposure were 10 μM and 5 μM for GF120918 and V-104, respectively. We examined reversal of resistance to etoposide, as 2008/MRP1 cells are highly resistant (20-fold) against this drug. V-104 reversed etoposide resistance of the 2008/MRP1 cells, but reversal was not complete, even at 5 μM (Table 2). Sensitivity of mock-transfected cells to etoposide also increased somewhat in the presence of V-104, presumably because of the inhibition of endogenous transporters. Inhibition of endogenous Pgp may also explain the effect found for GF120918 on etoposide resistance, as this effect did not clearly increase with GF120918 concentration.

### DISCUSSION

Specific inhibitors of drug transporters are potentially useful tools for the dissection of complex drug-resistance phenotypes and, eventually, for the treatment of patients with drug-resistant cancer. We have tested the specificity of three novel compounds, Pluronic L61, V-104 and GF120918, on three drug transporters, MDR1 Pgp, MRP1 and MRP2, in intact (transfected) cells. The transfected LLC-MDR1 cell line used in this study was selected with vincristine, whereas the MDCKII-derived cell lines were selected with geneticin (G-418). We therefore cannot formally exclude that

© 2000 Cancer Research Campaign

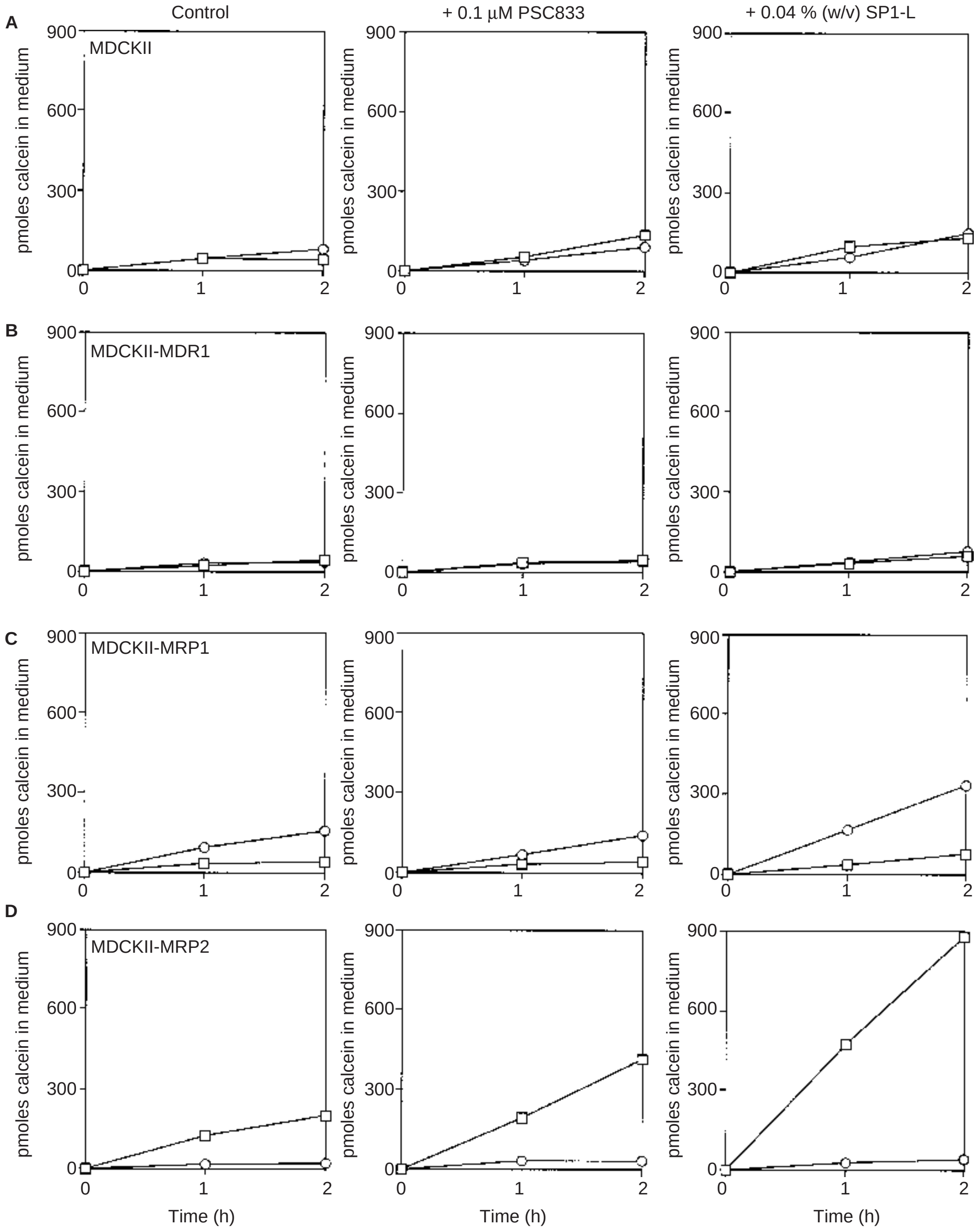

**Figure 3** Export of calcein by MDCKII cell monolayers. (**A, left graph**) MDCKII wild-type cells. At *t* = 0 calcein AM (1 μM) was added to both the apical and basolateral medium, and the amount of calcein appearing in the medium was measured using a fluorimeter. Samples were taken at *t* = 1 and *t* = 2 h. (**Middle and right graph**) Same as left graph, but the assay was performed in the presence of PSC833 (0.1 μM) or Pluronic L61 (0.04%), respectively. (**B–D**) Same as (**A**), but MDCKII-MDR1, MDCKII-MRP1 and MDCKII-MRP2 cells were tested, respectively. Squares: export to apical compartment. Circles: export to basolateral compartment these lines have developed some unknown mechanisms of drug resistance. We consider this, however, an unlikely possibility as untransfected cells never formed colonies after selection with vincristine or G-418, respectively. All three compounds completely inhibit MDR1 Pgp-mediated transport of vinblastine and daunorubicin at non-cytotoxic concentrations in our assays. GF120918 and V-104 have no effect on transport by MRP2. V-104 is a weak inhibitor of MRP1, but we only observed clear inhibition at drug concentrations close to cytotoxic levels. It is therefore unlikely that this drug will be useful for inhibiting MRP1 in human cancer. A high concentration (0.1%) of Pluronic L61 partially inhibited vinblastine transport by MRP2 (Figure 2), but had only a small effect on DNP-GS transport by MRP2. Our data also show that the interpretation of inhibitor studies can be complicated by the presence in the cells of other endogenous transporters with a high affinity for the inhibitor, the transport substrate used, or both. In the screening for inhibitors of MRP1 and MRP2, it is obviously important to use both organic anions and cytotoxic drugs as substrates, as inhibitors may have a differential effect on the transport of different classes of drugs.

 *© 2000 Cancer Research Campaign*

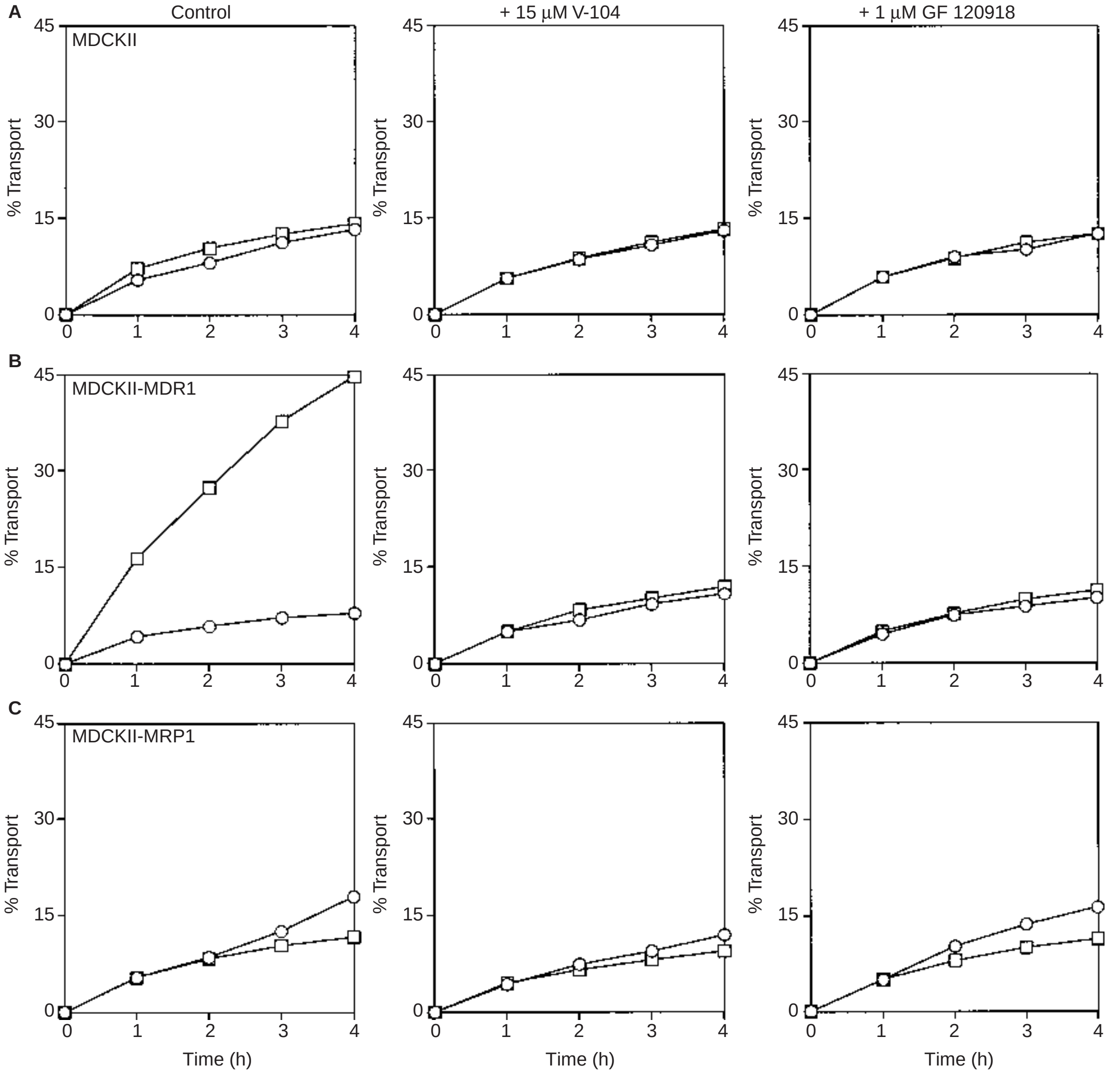

**Figure 4** Effect of V-104 and GF120918 on daunorubicin transport through polarized MDCKII cell monolayers. Transport was measured as described in Figure 2, but with [$^3$H]daunorubicin as substrate. (**A, left graph**) MDCKII cells. Transport in the presence of PSC833 (0.1 μM) alone. (**Middle and right graph**) Same as left graph, but in addition to PSC833, V-104 (15 μM) and GF120918 (1 μM) were added at *t* = 0. (**B,C**) Same as **A**, but MDCKII-MDR1 and MDCKII-MRP1 cells were tested, respectively. Squares: translocation from basolateral to apical. Circles: translocation from apical to basolateral.

Previous work has shown that P85, an analog of Pluronic L61, inhibits an apically localized drug transporter in CaCo-2 cells, most likely MDR1 Pgp (Batrakova et al, 1998). With transfected cell lines we show here that MDR1 Pgp is indeed inhibited by this class of compounds. The mechanism of action of the copolymer Pluronic L61 is not clear. It is not known whether it is a substrate for MDR1 Pgp (and therefore acts as a competitive inhibitor) or blocks MDR1 Pgp by another mechanism. Amphiphilic block copolymers have a low critical micelle concentration (cmc); the cmc for Pluronic L61 is 0.02% (w/v) (Batrakova et al, 1996). As we see a major inhibition of MDR1 Pgp below 0.02% (w/v) (Figure 1), we conclude that the monomeric form of Pluronic L61 is the effective species in inhibiting transport, in agreement with earlier suggestions by Miller et al (1997) and Batrakova et al (1998) for Pluronic analogues, and Nerurkar et al (1997) for other neutral surfactants. Our results show that the inhibitory effect of Pluronic L61 on Pgp is highly specific. We clearly show that Pluronic L61 does not increase the rate of passive diffusion of drug through the monolayer and that it has no clear effect on MRP1 or MRP2. It is therefore likely that Pluronic L61 directly binds to Pgp, like other inhibitors, but we have not verified this in a simple vesicular transport system, e.g. by vanadate-mediated nucleotide trapping (Szabo et al, 1998). Very recently, Miller et al (1999) suggested that Pluronic L61 and P85 do inhibit MRP1 in a human pancreatic adenocarcinoma cell line. These authors studied the accumulation of fluorescein and observed some increased intracellular accumulation in the presence of various Pluronic analogues. The discrepancy with our data may be explained by the presence of other organic anion transporters than MRP1 in pancreatic adenocarcinoma cells; one of these might be sensitive towards Pluronic analogues. Our data clearly indicate that in MDCKII cells, neither MRP1 nor MRP2 are inhibited significantly by Pluronic L61. Firstly, DNP-GS export by MRP1 or MRP2 was only slightly impaired (Table 1) and, secondly, vinblastine

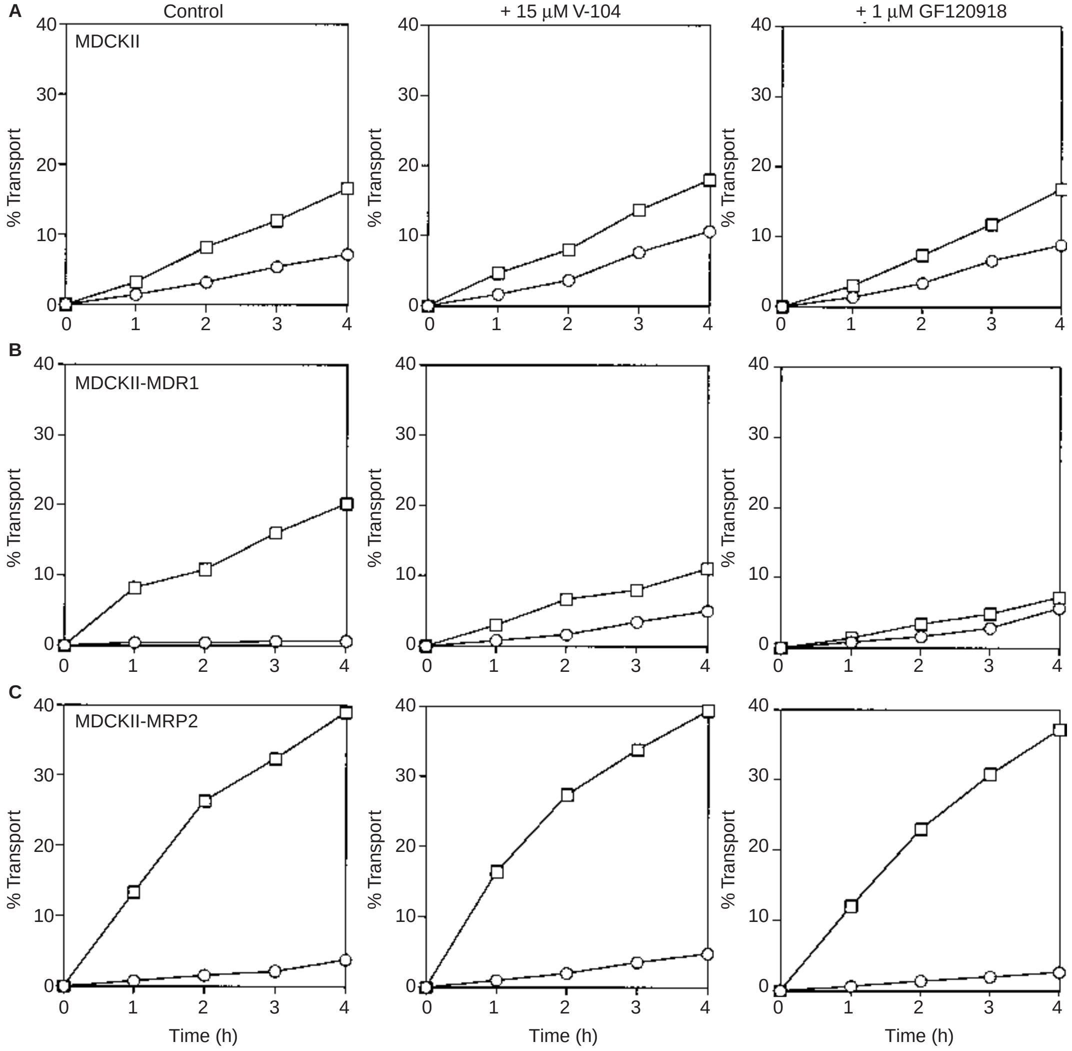

**Figure 5** Effect of V-104 and GF120918 on vinblastine transport through polarized MDCKII monolayers. Transport was measured as in Figure 4, but with [$^3$H]vinblastine as substrate.

**Table 2** Growth inhibition of 2008/MRP1 and mock transfected 2008/pCMV-neo cells by etoposide

| | **2008/pCMV-neo** $IC_{50}$ **(nmol)** | **2008/MRP1** $IC_{50}$ **(nmol)** |
|---|---|---|
| Etoposide | 0.42 ± 0.06 (1.0) | 8.84 ± 1.44 (20) |
| Etoposide + 1.0 μM GF120918 | 0.36 ± 0.00 (0.9) | 9.77 ± 5.24 (27) |
| Etoposide + 2.5 μM GF120918 | 0.31 ± 0.03 (0.7) | 4.27 ± 0.80 (14) |
| Etoposide + 5.0 μM GF120918 | 0.31 ± 0.01 (0.7) | 6.98 ± 1.86 (23) |
| Etoposide + 1.0 μM V-104 | 0.34 ± 0.02 (0.8) | 5.00 ± 2.17 (15) |
| Etoposide + 2.5 μM V-104 | 0.23 ± 0.00 (0.5) | 2.95 ± 0.87 (13) |
| Etoposide + 5.0 μM V-104 | 0.30 ± 0.11 (0.7) | 1.46 ± 0.17 (5) |

$IC_{50}$s are means of an experiment performed in triplicate. Numbers in parentheses represent resistance factors (relative to 2008/pCMV-neo incubated with etoposide alone for 2008/pCMV-neo cells; relative to the identically treated 2008/pCMV-neo cells for 2008/MRP1 cells).

 *© 2000 Cancer Research Campaign*

transport by MRP2 was only weakly affected at the highest concentration of Pluronic L61 tested (Figure 2). We observed stimulation of calcein export by MRP1 and MRP2 in the presence of Pluronic L61 (Figure 3), probably caused by the efficient inhibition of canine Pgp by this compound. As PSC833 could only partially mimic this effect, PSC833 cannot block calcein AM transport by Pgp completely, whereas Pluronic L61 can, or there is another endogenous transporter of calcein AM inhibited by Pluronic L61 but not by PSC833.

It is remarkable that many of the reversal agents that efficiently block MDR1 Pgp, like PSC833, GF120918 and Pluronic L61, have little or no effect on MRP1 and MRP2 (e.g Böhme et al, 1993; Wallstab et al, 1999). It has been shown that both GF120918 and PSC833 are competitive inhibitors that probably bind with high affinity to the substrate binding site(s) of MDR1 Pgp (Smith et al, 1998; Wallstab et al, 1999). Obviously the drug binding sites of MDR1 Pgp have a higher affinity for hydrophobic inhibitors than the drug binding sites of MRPs. This could be due to a difference in transport mechanisms. Whereas MDR1 Pgp does not require any co-factor for the transport of hydrophobic drugs, transport by MRP1 and MRP2 of these drugs is associated with the export of reduced glutathione (Rappa et al, 1997; Loe et al, 1998; Evers et al, 2000). The compounds that we used here are all relatively hydrophobic and not negatively charged. They may therefore require GSH for binding to MRPs, and this may result in a relatively low affinity for the transporter.

Most high-affinity substrates for MRP1 and MRP2 found thus far are organic anions with a substantial hydrophobic moiety and one, but preferably two, negative charge(s). Examples are leukotriene $C_4$, *S*-decylglutathione and the leukotriene $D_4$ antagonist MK571 (Loe et al, 1996; Keppler et al, 1998). These substrates do not readily enter cells and they therefore do not provide obvious lead compounds for drug development. As it seems unlikely that more membrane-permeable compounds can be found with high affinity for MRP, good inhibitors will have to be made as prodrugs in which the charged moiety is shielded and can be converted into active drug in the cell, like CDNB or calcein AM.

### ACKNOWLEDGEMENTS

We thank Dr V Alakhov (Supratek Pharma Inc., Canada), and Dr R Mashal (Vertex Pharmaceuticals, USA) for critically reading the manuscript and for providing Pluronic L61 and V-104, respectively. This work has been supported by the Dutch Cancer Society (research grants NK194–775 and NK198–1794).

### REFERENCES

Bakos E, Evers R, Szakacs G, Tusnad, GE, Welker E, Szabo K, de Haas M, van Deemter L, Borst P, Varadi A and Sarkadi B (1998) Functional multidrug resistance protein (MRP1) lacking the N-terminal transmembrane domain. *J Biol Chem* **273**: 32167–32175

Batrakova EV, Han HY, Miller DW and Kabanov AV (1998) Effects of pluronic P85 unimers and micelles on drug permeability in polarized BBMEC and Caco-2 cells. *Pharm Res* **15**: 1525–1532

Batrakova EV, Dorodnych TY, Klinskii EY, Kliushnenkova EN, Hemchukova OB, Goncharova ON, Arjakov SA, Alakhov VY and Kabanov AV (1996) Anthracycline antibiotics non-covalently incorporated into the block copolymer micelles: in vivo evaluation of anti-cancer activity. *Br J Cancer* **74**: 1545–1552

Böhme M, Büchler M., Müller M and Keppler D (1993) Differential inhibition of cyclosporins of primary-active ATP-dependent transporters in the hepatocyte canalicular membrane. *FEBS Lett* **333**: 193–196

Chen ZS, Kawabe T, Ono M, Aoki S, Sumizawa T, Furukawa T, Uchiumi T, Wada M, Kuwano M and Akiyama SI (1999) Effect of multidrug resistance-reversing agents on transporting activity of human canalicular multispecific organic anion transporter. *Mol Pharmacol* **56**: 1219–1228

Cole SPC and Deeley RG (1998) Multidrug resistance mediated by the ATP-binding cassette transporter protein MRP. *Bioassays* **20**: 931–940

Cole SPC, Bhardwaj G, Gerlach JH, Mackie JE, Grant CE, Almquist KC, Stewart AJ, Kurz EU, Duncan AMV and Deeley RG (1992) Overexpression of a transporter gene in a multidrug-resistant human lung cancer cell line. *Science* **258**: 1650–1654

Cui Y, König J, Buchholz U, Spring H, Leier I and Keppler D (1999) Drug resistance and ATP-dependent conjugate transport mediated by the apical multidrug resistance protein, MRP2, permanently expressed in human and canine cells. *Mol Pharmacol* **55**: 929–937

Evers R, Cnubben WHP, Wijnholds J, van Deemter C, van Bladeren P and Borst P (1997) Transport of glutathione prostaglandin A conjugates by the multidrug resistance protein 1. *FEBS Lett* **419**: 112–116

Evers R, de Haas M, Sparidans R, Beijnen J, Wielinga PR, Lankelma L and Borst P (2000) Vinblastine and sulfinpyrazone export by the multidrug resistance protein MRP2 is associated with glutathione export. *Br J Cancer* **83**: 375–383

Evers R, Zaman GJR, van Deemter L, Jansen H, Calafat J, Oomen LCJM, Oude Elferink RPJ, Borst P and Schinkel AH (1996) Basolateral localization and export activity of the human multidrug resistance-associated protein in polarized pig kidney cells. *J Clin Invest* **97**: 1211–1218

Evers R, Kool M, van Deemeter L, Janssen H, Calafat J, Oomen LCJM, Paulusma CC, Oude Elferink RPJ, Baas F, Schinkel AH and Borst P (1998) Drug export activity of the human canalicular multispecific organic anion transporter in polarized kidney MDCK cells expressing *cMOAT* (*MRP2*) cDNA. *J Clin Invest* **101**: 1310–1319

Feller N, Broxterman HJ, Wähler DCR and Pinedo HM (1995) ATP-dependent efflux of calcein by the multidrug resistance protein (MRP): no inhibition by intracellular glutathione depletion. *FEBS Lett* **368**: 385–388

Fisher GA and Sikic BI (1995) Clinical studies with modulators of multidrug resistance. *Hematol Oncol Clin North Am* **9**: 363–382

Germann UA, Shlyakhter D, Mason VS, Zelle RE, Duffy JP, Galullo V, Armistead DM, Saunders JO, Boger J and Harding MW (1997*a*) Cellular and biochemical characterization of VX-710 as a chemosensitizer: reversal of P-glycoprotein-mediated multidrug resistance in vitro. *Anticancer Drugs* **8**: 125–140

Germann UA, Ford PJ, Shlyakhter D, Mason VS and Harding MW (1997*b*) Chemosensitization and drug accumulation effects of VX710, verapamil, cyclosporin A, MS209 and GF120918 in multidrug resistant HL60/ADR cells expressing the multidrug resistance-associated protein MRP. *Anticancer Drugs* **8**: 141–155

Gottesmann MM, Hrycyna CA, Schoenlein PV, Germann UA and Pastan I (1995) Genetic analysis of the multidrug transporter. *Annu Rev Genet* **29**: 607–649

Hirohashi T, Suzuki H and Sugiyama Y (1999) Characterization of the transport properties of cloned rat multidrug resistance protein 3 (MRP3). *J Biol Chem* **274**: 15181–15185

Holló Z, Homolya T, Hegedus T and Sarkadi B (1996) Transport properties of the multidrug resistance-associated protein (MRP) in human tumour cells. *FEBS Lett* **383**: 99–104

Homolya L, Holló Z, Müller M, Mechetner EB and Sarkadi B (1996) A new method for a quantitative assessment of P-glycoprotein-related multidrug resistance in tumour cells. *Br J Cancer* **73**: 849–855

Hooijberg JH, Broxterman HJ, Kool M, Assaraf YG, Peters GJ, Noordhuis P, Scheper RJ, Borst P, Pinedo HM and Jansen G (1999) Antifolate resistance mediated by the multidrug resistance proteins MRP1 and MRP2. *Cancer Res* **59**: 2532–2535

Hyafil F, Vergely C, Du Vignaud P and Grand-Perret T (1993) In vitro and in vivo reversal of multidrug resistance by GF120918, an acridonecarboxymide derivative. *Cancer Res* **53**: 4595–4602

Jedlitschky G, Leier I, Buchholz U, Hummel-Eisenbeiss J, Burchel B and Keppler D (1997) ATP-dependent transport of bilirubin glucuronides by the multidrug resistance protein MRP1 and its hepatocyte canalicular isoform MRP2. *Biochem J* **327**: 305–310

Juliano RL and Ling V (1976) A surface glycoprotein modulating drug permeability in Chinese hamster ovary cell mutants. *Biochem Biophys Acta* **455**: 152–162

Keppler D, Jedlitschky G and Leier I (1998) Transport function and substrate specificity of multidrug resistance proteins. *Methods Enzymol* **292**: 607–616

Kool M, van der Linden M, de Haas M, Baas F and Borst P (1999*a*) Expression of human MRP6, a homologue of the multidrug resistance protein gene MRP1, in tissues and cancer cells. *Cancer Res* **59**: 175–182

Kool M, van der Linden M, de Haas M, Scheffer GL, de Vree ML, Smith AJ, Jansen G, Peters GJ, Ponne N, Scheper RJ, Oude Elferink RPJ, Baas F and Borst P

© 2000 Cancer Research Campaign

(1999*b*) MRP3, a new organic anion transporter able to transport anti-cancer drugs. *Proc Natl Acad Sci USA* **96**: 6914–6919

Kool M, de Haas M, Scheffer GL, Scheper RJ, van Eijk MJT, Juijn JA, Baas F and Borst P (1997) Analysis of expression of cMOAT (MRP2), MRP3, MRP4, and MRP5, homologs of the multidrug resistance-associated protein gene (MRP1), in human cancer cell lines. *Cancer Res* **57**: 3527–3547

Leier I, Jedlitschky G, Buchholz U, Cole SPC, Deeley RG and Keppler D (1994) The MRP gene encodes an ATP-dependent export pump for leukotriene C4 and structurally related conjugates. *J Biol Chem* **269**: 27807–27810

Loe DW, Almquist KC, Deeley R and Cole SPC (1996) Multidrug resistance protein (MRP)-mediated transport of leukotriene C4 and chemotherapeutic agents in membrane vesicles. *J Biol Chem* **271**: 9675–9682

Loe DW, Deeley RG and Cole SPC (1998) Characterization of vincristine transport by the M(r) 190 000 multidrug resistance protein (MRP): evidence for cotransport with reduced glutathione. *Cancer Res* **58**: 5130–5136

Masuda M, I'izuka Y, Yamazaki M, Nishigaki R, Kato Y, Ni'inuma K, Suzuki H and Sugiyama Y (1998) Methotrexate is excreted into the bile by canalicular multispecific organic anion transporter in rats. *Cancer Res* **57**: 3506–3510

Miller DW, Batrakova EV and Kabanov AV (1999) Inhibition of multidrug resistance-associated protein (MRP) functional activity with pluronic block copolymers. *Pharmaceutical Res* **16**: 396–401

Miller DW, Batrakova EV, Waltner TO, Alakhov VY and Kabanov AV (1997) Interactions of pluronic block copolymers with brain microvessel endothelial cells: evidence of two potential pathways for drug absorption. *Bioconjugate Chem* **8**: 649–657

Müller M, Meijer C, Zaman GJR, Borst P, Scheper RJ, Mulder NH, de Vries EGE and Janssen PLM (1994) Overexpression of the gene encoding the multidrug resistance-associated protein results in increased ATP-dependent glutathione *S*-conjugate transport. *Proc Natl Acad Sci USA* **91**: 8822–8826

Nerurkar MM, Ho NFH, Burton PS, Vidmar TJ and Borchardt RT (1997) Mechanistic roles of neutral surfactants on concurrent polarized and passive membrane transport of a model peptide in Caco-2 cells. *J Pharm Sci* **86**: 813–821

Oude Elferink RPJ, Bakker CTM and Jansen PLM (1993) Glutathione-conjugate transport by human colon adenocarcinoma cells (Caco-2 cells). *Biochem J* **290**: 759–764

Rappa J, Lorico A, Flavell RA and Sartorelli AC (1997) Evidence that the multidrug resistance protein (MRP) functions as a co-transporter of glutathione and natural product toxins. *Cancer Res* **57**: 5232–5237

Rowinsky EK, Smith L, Wang YM, Chaturvedi P, Villalona M, Campbell E, Aylesworth C, Eckhardt SG, Hammond L, Kraynak M, Drengler R, Stephenson J, Harding MW and Von Hoff DD (1998) Phase I and pharmacokinetic study of paclitaxel in combination with biricodar, a novel agent that reverses multidrug resistance conferred by overexpression of both MDR1 and MRP. *J Clin Oncol* **16**: 2964–2967

Sarkadi B and Müller M (1997) Search for specific inhibitors of multidrug resistance in cancer. *Semin Cancer Biol* **8**: 171–182

Schinkel AH, Wagenaar E, van Deemter L, Mol CAAM and Borst P (1995) Absence of the mdr1a P-glycoprotein in mice affects the tissue distribution and pharmacokinetics of dexamethasone, digoxin, and cyclosporin A. *J Clin Invest* **96**: 1698–1705

Schuetz JD, Conelly MC, Sun D, Paibir SG, Flynn PM, Srinivas RV, Kumar A and Fridland A (1999) MRP4: A previously unidentified factor in resistance to nucleoside based antiviral drugs. *Nat Med* **5**: 1048–1051

Smith AJ, Mayer U, Schinkel AH and Borst P (1998) Availability of PSC833, a substrate and inhibitor of P-glycoprotein, in various concentrations of serum. *J Natl Cancer Inst* **90**: 1161–1166

Szabo K, Welker E, Bakos E, Muller M, Roninson I, Varadi A and Sarkadi B (1998) Drug stimulated nucleotide trapping in the human multidrug transporter MDR1. Cooperation of the nucleotide binding domains. *J Biol Chem* **273**: 10132–10138

Twentyman PR and Bleehen NM (1991) Resistance modification by PSC833, a novel non-immunosuppressive cyclosporin. *Eur J Cancer* **27**: 1639–1642

Venne A, Li S, Mandeville R, Kabanov A and Alakhov V (1996) Hypersensitizing effect of pluronic L61 on cytotoxic activity, transport, and subcellular distribution of doxorubicin in multiple drug-resistant cells. *Cancer Res* **56**: 3626–3629

Wallstab A, Koester M, Böhme M and Keppler D (1999) Selective inhibition of MDR1 P-glycoprotein-mediated transport by the acridone carboxamide derivative GG918. *Br J Cancer* **79**: 1053–1060

Yanagisawa T, Newman A, Coley H, Renshaw J, Pinkerton CR and Pritchard-Jones K (1999) Biricodar (VX-710; Incel): a selective chemosensitizer in neuroblastoma. *Br J Cancer* **80**: 1190–1196

 *© 2000 Cancer Research Campaign*
